# A Comprehensive Method to Evaluate the Usability of Virtual Reality Headset Devices for Industrial Applications

**DOI:** 10.3390/s26134038

**Published:** 2026-06-25

**Authors:** Marco Cirelli, Alessio Cellupica, Pier Paolo Valentini, Luigi Cinque, Marco Raoul Marini

**Affiliations:** 1 Department of Enterprise Engineering “Mario Lucertini”, University of Rome Tor Vergata, Via del Politecnico 1, 00133 Rome, Italy; marco.cirelli@uniroma2.it (M.C.); alessio.cellupica@uniroma2.it (A.C.); 2 Department of Computer Science, Sapienza University of Rome, Via Salaria 113, 00198 Rome, Italy; luigi.cinque@uniroma1.it (L.C.); marcoraoul.marini@uniroma1.it (M.R.M.)

**Keywords:** virtual reality, VR headset, usability methodology, industrial virtual environment, user-centered evaluation, interaction performance, assembly simulation, ergonomic assessment

## Abstract

The increasing adoption of virtual reality for industrial tasks such as virtual assembly, inspection, and operator training necessitates a standardized approach for evaluating and selecting appropriate hardware. This paper addresses this need by introducing a comprehensive methodology to assess the usability of commercially widespread virtual reality headsets specifically for industrial applications with hand-held controllers. We conducted a large-scale comparative study involving five leading headsets (HTC VIVE Pro 1 and 2, HTC VIVE XR Elite, Meta Quest Pro, and Meta Quest 3) and 60 demographically balanced participants. The evaluation was based on a protocol of 15 distinct tasks designed to measure performance in near and far-field object manipulation, interaction fidelity, visual clarity, ergonomics, and long-term comfort. By combining quantitative Key Performance Indicators with subjective user feedback and rigorous inferential statistical analysis, our findings reveal significant performance disparities among the devices. The results demonstrate that, while certain headsets excel in high-precision tracking for assembly tasks, others offer superior comfort, visual quality, and ease of use for inspection and prolonged sessions. Ultimately, this study concludes that no single headset is universally superior; the optimal choice is highly task-dependent. The proposed methodology provides a robust, evidence-based framework to guide industries in making informed virtual reality hardware selections tailored to their specific needs.

## 1. Introduction

In recent years, interactive virtual reality (VR) and augmented reality (AR) technologies have experienced a rapid expansion across both scientific and industrial domains. This growth has been fueled by the increasing availability of diverse hardware devices, the development of tools for designing immersive and interactive environments, and the accessibility of high-performance computing platforms capable of supporting real-time simulations. These factors have significantly lowered the barriers to the adoption of VR, enabling its integration into a wide spectrum of applications ranging from scientific visualization and education to product development and training [1,2,3]. In the industrial context in particular, VR has emerged as a versatile tool for enhancing productivity, safety, and efficiency. The recent literature highlights its potential across different sectors, underscoring the role of VR in improving complex workflows and supporting decision-making. Three prominent classes of applications have gained considerable attention. First, assembly training applications are supported by VR to immerse operators in realistic scenarios, allowing them to practice complex procedures without the risks and costs associated with physical prototypes [4,5,6]. Some methodologies of virtual assembly are shared with AR implementations or even use the same devices. In particular, [7,8,9] introduced and refined methods for interactive virtual assembly in augmented and virtual reality, including natural hand interaction and physics-based manipulation. The present study adopts the same interaction logic (e.g., pick, rotate, insert) but scales it to a comparative usability benchmark across multiple commercial headsets. Second, visual inspection tasks have been supported by VR-based solutions that enable detailed examination of components, defect detection, and quality assurance within controlled environments [10,11,12]. And also, medical training represents one of the most promising areas of VR adoption, where immersive simulations support surgical rehearsal, anatomy exploration, and skill acquisition in safe yet highly realistic conditions [13,14,15]. Within this domain, prior work by some of the authors has contributed methodological advances that are directly relevant to the present usability study. Avola et al. [16] developed an immersive VR endoscopic prototype that captured fine motor actions and task completion times, demonstrating how quantitative performance metrics can be extracted from VR simulations. Avola et al. [17] introduced VRheab, a motor rehabilitation system based on recurrent neural networks, which relied on repeated user trials and objective logging of movement accuracy. Avola et al. [18] proposed a low-cost full-body rehabilitation framework using serious games, emphasizing the use of weighted trial averaging to reduce learning and fatigue effects. Although these studies addressed clinical applications, they established practical protocols for collecting and processing user performance data in VR—specifically, the use of multiple repetitions per task and the handling of outlier trials—which we directly adapt in our KPI computation (Equations (1) and (2)). Thus, these self-citations are motivated by methodological continuity, not by mere self-reference. Together, these examples demonstrate the cross-disciplinary relevance of VR for industrial and professional training, while also pointing to its critical role in supporting human-centered tasks. Another rapidly growing domain where interactive VR simulations are gaining impetus is that of industrial digital twins. The coupling of digital twins (virtual replicas of physical assets updated in real time) with immersive VR environments enables not only advanced data visualization but also direct interaction with the digital model for tasks such as monitoring, fault diagnosis, layout validation, and the simulation of complex operational procedures. Several studies have highlighted how VR can serve as a spatial and collaborative interface for digital twins, enhancing the understanding of multiphysics phenomena and facilitating shared decision-making among heterogeneous teams (engineering, production, maintenance). Recent industrial examples further demonstrate concrete applications, ranging from engineering reviews and project validation to solutions enabling remote inspection and maintenance of critical assets through their digital replicas [19,20,21]. Chheang et al. [19] showed how collaborative virtual reality can support part inspection in additive manufacturing by overlaying digital twins onto physical components. Yang et al. [20] developed a live digital twin system with VR interfaces for immersive manufacturing monitoring and control. Mahdi et al. [21] proposed an OPC UA-based architecture for wire arc additive manufacturing, addressing real-time data synchronization between physical and virtual assets. These implementations clearly showcase the potential of VR as an interaction layer for digital twins, while also underscoring the importance of comparative evaluations of hardware in terms of latency, tracking accuracy, and usability factors that ultimately determine the effectiveness of VR–digital twin integration in operational industrial settings.

In this context, three recent works by the authors have established methodological foundations that directly inform the present study. Cellupica et al. [22] developed an interactive digital twin framework for training users in the use of a sensorized upper-limb prosthesis within a VR environment, introducing a reusable architecture for real-time sensor data integration and haptic feedback. Cirelli et al. [23] demonstrated a real-time interactive digital twin for structural dynamics, combining reduced-order modeling with augmented reality to allow users to manipulate and interrogate a mechanical component under impulse loads, highlighting the importance of low-latency interaction and physics fidelity. Cirelli et al. [24] extended this approach to augmented reality for structural digital twins, focusing on real-time visualization and user-driven exploration of reduced-order models. Together, these studies identify key requirements for immersive digital twin interfaces, such as low tracking latency, stable controller interaction, and visual clarity, which directly depend on the choice of VR hardware. The present study therefore builds on these prior works by shifting the focus from feasibility of individual digital twin applications to a systematic, comparative evaluation of commercial VR headsets under controlled, task-oriented conditions. From the literature, different contributions can be identified: reviews discussing architectural requirements and fidelity levels of digital twins; applied studies integrating VR for real-time control and monitoring (e.g., mining or additive manufacturing contexts); and research evaluating the role of immersive interaction in reducing diagnosis time and improving the quality of operational decisions. These works also emphasize practical challenges, including real-time data synchronization, network latency, visualization of large CAD models within VR scenes, and the need for user interfaces that make dynamic twin data explicit, all of which directly affect hardware selection and user experience design. Recent industrial examples further demonstrate concrete applications, ranging from engineering reviews and project validation to solutions enabling remote inspection and maintenance of critical assets through their digital replicas [25,26,27]. These implementations clearly showcase the potential of VR and AR as an interaction layer for digital twins, while also underscoring the importance of comparative evaluations of hardware in terms of latency, tracking accuracy, and usability factors that ultimately determine the effectiveness of VR–digital twin integration in operational industrial settings. Despite these advances, significant challenges remain in the implementation of realistic and interactive VR scenes. Achieving a convincing sense of immersion requires not only high-fidelity graphical rendering but also low-latency interaction, ergonomic usability, and seamless integration of hardware and software. These requirements impose constraints on both device selection and system design, often leading practitioners to choose VR headsets and controllers based primarily on technical specifications such as resolution, refresh rate, or tracking capabilities. However, such specifications do not always translate into optimal user interaction or usability in practice. Moreover, many VR environments are developed in a device-agnostic manner, which ensures portability across platforms but frequently results in suboptimal performance and interaction quality [28,29,30]. This gap between hardware capabilities and actual user experience underscores the need for systematic evaluations that extend beyond datasheets and specifications. Recent contributions have started to address the need for systematic evaluations of XR hardware in industrial contexts. Barve and De Amicis [31] conducted one of the first user-centered validations of a low-cost VR head-mounted display (HMD) harvester simulator, quantifying cognitive load, usability, and simulator sickness with established instruments. Gornall et al. [32] provided a systematic review of XR applications in Construction 4.0, mapping which XR modes, devices, and graphics engines are most prevalent, and identifying implementation barriers. Malva et al. [33] performed a qualitative, criteria-based comparative analysis of eleven commercially available AR/MR devices for physical asset management, evaluating them against operational requirements such as ergonomics, display characteristics, and spatial mapping performance. Together, these studies underscore the growing interest in evidence-based hardware assessment for professional and industrial XR deployments, yet none of them offers a standardized, task-oriented benchmarking methodology focused on controller-based VR interaction for assembly, inspection, and training operations, the specific gap addressed by the present work. To fill this gap, the present study introduces a methodological framework with several novel elements beyond a simple device comparison. First, we propose a composite performance metric that normalizes task execution times and accuracy scores onto a common scale, making the comparison fair and reproducible across different headsets. Second, our task set is systematically designed to isolate the fundamental interaction primitives underlying industrial operations, such as grasping, rotating, inserting, button pressing, and throwing, following established ergonomic standards. Third, we adopt a weighted scoring scheme to reduce the influence of learning and fatigue effects across repeated trials, improving the reliability of the collected data. Finally, the entire evaluation pipeline, from task definition to statistical analysis (including repeated measures ANOVA and post hoc comparisons), is presented as a reusable benchmark for future assessments of VR hardware. Taken together, these elements constitute a transferable usability benchmarking methodology for VR headsets in industrial contexts, not merely a one-time device comparison. The objective of the present study is to address this gap by providing a comparative analysis of leading VR hardware devices with a focus on user interaction and general usability, evaluated through quantitative metrics rather than qualitative impressions. This is unlike prior work, which often relies on subjective assessments, single-device studies, or generalized specifications [34,35,36,37]; for example, Kamm et al. focused on the feasibility of a single headset for dexterity training in multiple sclerosis, Khorasani et al. compared interaction styles without cross-device analysis, Chang et al. reviewed VR sickness measurement methods, and Kaminska et al. provided general usability guidelines without quantitative KPIs. The present research employs a set of application-driven tests designed specifically to reflect industrial use cases, comparing five commercial headsets through objective metrics and inferential statistics. The analysis focuses on the interaction mediated through hand-held controllers, which remain the most widespread and reliable mode of input in industrial VR applications. Gesture recognition, while increasingly supported by many devices, is still heavily dependent on software libraries and contextual conditions, making standardized comparisons more complex at present. By narrowing the scope to controller-based interaction, the study ensures reproducibility and comparability across platforms, while also providing insights that are directly relevant to the majority of current industrial VR applications. Due to the exploratory nature of this work and the absence of prior direct comparisons among the selected devices, no directional hypotheses are formulated. Instead, the study aims to systematically collect quantitative and qualitative usability data, providing a foundation for future hypothesis-driven research.

### 1.1. Scope and Contextual Boundaries

The proposed benchmarking protocol is built on a set of deliberate methodological choices that ensure reproducibility and generalizability. The tests were conducted in a single laboratory environment with controlled lighting and background noise (Section 3). While this may not replicate all real-world industrial conditions, it eliminates external variability and allows direct comparison of devices under identical physical conditions. A single software stack (Unity XR Interaction Toolkit, v.2.5) and a platform-agnostic virtual environment were used across all headsets (Section Description of the Tested Devices and Objective). This guarantees that differences in performance are not due to software optimization but to the hardware itself, making the benchmark transferable to other engines or devices. Participants were typical users with mixed levels of VR experience (Section 3), reflecting real industrial training scenarios where operators are rarely VR experts. The weighted averaging of trials (Equation (1)) and the threshold-based KPI (Equation (2)) further reduce the influence of individual outliers, making the results robust across different user aptitudes. Only five headsets from two manufacturers were included (Section Description of the Tested Devices and Objective), but the task set and KPI framework are device-agnostic and can be directly applied to any future headset. The use of primitive geometries (cubes, spheres, simple prisms) instead of complex CAD models is not a limitation but a controlled choice to isolate low-level hardware responsiveness (tracking, button latency, visual clarity) from confounding factors such as polygon count or scene complexity. Consequently, the benchmark serves as a foundational, generalizable tool for evaluating VR headsets for controller-based industrial assembly and inspection tasks. Extensions to large CAD models, multi-user collaboration, or live digital twin synchronization are orthogonal and can be built upon this framework in future work.

### 1.2. Paper Organization

The paper is organized as follows. The first part is dedicated to the presentation of the technical specifications of the selected devices. The second part of the paper focuses on the methodological development of a VR usability evaluation approach. Specifically, it introduces a practical, metric-based usability testing protocol designed for commercial VR devices used in industrial assembly and inspection scenarios. By combining objective Key Performance Indicators (KPIs) with subjective user feedback across 15 representative tasks, the proposed method provides comparative insights that are currently lacking in the literature. To ensure a representative sample of typical industrial operators, the evaluations were performed by participants with diverse professional backgrounds and no prior specialization in VR technologies; all user interactions and subjective feedback were systematically recorded. The final section is dedicated to the summarization and discussion of the results.

## 2. Technological Review

In this section, the technological review is conducted, describing the involved tools, the proposed tasks, the participants’ details, and the metrics.

### Description of the Tested Devices and Objective

In the field of virtual assembly and virtual inspection, the user is required to perform certain actions or recognize system issues through sensory perceptions (sight, hearing, touch, etc.) [1]. During the assembly of a complex system, the user is required to move and rotate objects or place them into designated slots, ensuring stable couplings. Movements of this kind require dexterity and accuracy to avoid potential assembly errors. In virtual assembly, this level of accuracy is required for a realistic experience. In this way, it is possible to train users to perform the operations they need to execute on physical objects in a virtual environment or improve procedures and tasks. Virtual reality devices, in general, comprised a head-mounted display and a pair of hand controllers. As well as the virtual environment being important, using the right device for the right application could enhance the user experience and interaction. For this purpose, it is important to have an assessment and comparison of both graphical performance and user tracking capabilities.

There are many consumer devices available on the market, with different hardware characteristics, that can be used for virtual reality (VR) interfaces. Not all devices are optimized to perform certain operations; therefore, a usability analysis is necessary to highlight their strengths and weaknesses. For this study, five commercial devices have been tested and compared in terms of virtual assembly and inspection usability. Three are manufactured by the HTC company (HTC Corporation, New Taipei, Taiwan): HTC VIVE Pro 1, HTC VIVE Pro 2, and HTC VIVE XR Elite; and two by Meta (Meta Platforms, Inc., 1 Meta Way, Menlo Park, CA, USA): Meta Quest 3 and Meta Quest Pro.

To ensure fair comparison of device usability independently of computational power, we tested all headsets under the following controlled conditions: (i) all devices were connected via wired PC streaming to the same high-performance workstation, guaranteeing identical rendering resources; (ii) each headset operated at its native refresh rate and resolution (e.g., 120 Hz for Meta Quest 3 and HTC VIVE Pro 2, 90 Hz for the others) to preserve the manufacturer’s intended visual experience (the resolutions are those reported in the comparative Table 1); (iii) the same virtual environment and interaction logic (Unity XR Interaction Toolkit) were used across all devices without modification. These assumptions are not hypotheses about expected outcomes, but rather control premises that ensure the validity of subsequent comparisons. While these differences complicate direct attribution of performance to a single hardware parameter, they reflect the holistic usability of each device, which is the primary focus of this study. It is important to highlight that mixed reality capabilities (e.g., passthrough, spatial anchoring, real-world overlays) are outside the scope of this study, which focuses exclusively on controller-based VR interaction.

## 3. Description of Usability Tests in Human–System Interaction

The concept of usability, defined according to the ISO 9241 standard of 1998 and integrated by the ISO/IEC 25010 standard, includes the effectiveness, efficiency, and satisfaction of specific users in achieving specific goals in a specific context of use. Usability testing is a technique that evaluates a product overall by testing it on potential users, providing direct input on their system usage. This type of test aims to verify if the product meets the intended goals and requirements. The state of the art presents many cases of usability analysis about virtual reality that could serve as guidelines. Kamińska et al. [37] provided some procedures to follow when conducting a usability analysis of a VR environment, outlining the steps and actions to be taken or avoided during the analysis. To optimize tools and virtual environments for their use in the industrial field, various aspects are considered in other examples of VR usability analysis, including small movements, basic actions, the level of immersion, and the user’s sickness. Nenna et al. [38] identified the level of user fatigue when using physical movements to control robotic systems in a virtual environment. The primary focus of this analysis was on two main questions: how interactive features of VR affect user performance and workload, and the sensitivity of various eye parameters in monitoring users’ vigilance and workload during the task. Meanwhile, Luo et al. [39] investigated the impact of scenario fidelity and interaction in VR-based forklift safety training, with a focus on usability, aiming to train users to follow safety protocols in an immersive environment. In the industrial sector, virtual reality is primarily employed for worker training in two main areas of focus: virtual assembly and virtual inspection. In relation to virtual assembly, various aspects of basic movements have been investigated to pinpoint strengths and weaknesses for personnel training. For example, Roldán et al. [40] introduce a training system for industrial operators in assembly tasks that leverages virtual reality. Users are required to perform assembly tasks in a virtual reality environment and then replicate the same assembly in the real world. Other researchers [41,42,43] have used virtual reality to assess small movements; meanwhile, Wolfartsberger et al. [3] explored whether VR-supported training leads to an increase in learning success compared to traditional on-the-job training accompanied by a tutor, focusing on simplified assembly processes. Also, Dimitrokalli et al. [44] focused on the collaboration between humans and robots through virtual reality training to optimize assembly processes during the production process. Kamm et al. [34] proposed a virtual reality training program followed by a usability and feasibility analysis. It was conducted on patients with multiple sclerosis who were subjected to performing the following exercises in the virtual environment: catching apples, finger circling, bending/stretching fingers, pinch grip, tracing shapes, wrist rotation. Other studies [30,35,45,46] show how virtual reality can influence the training process for specific assembly cases. Recently, ref. [22] has discussed a complete methodology and the supporting algorithms to develop a virtual reality environment to train the use of a sensorized upper-limb prosthesis targeted at amputees. In ref. [9], the OAF-GAF methodology is implemented in an augmented reality environment for tree-hole assembly. In the context of virtual inspection, potential frameworks have been devised to assist workers in carrying out maintenance processes in the industrial field. The state of the art presents various case studies; for instance, Wang et al. [47] introduced a framework for specific techniques that can be implemented in remote infrastructure inspections, thus identifying potentially damaged areas and preventing possible causes of breakdown. Other researchers [48,49,50] showed how modern VR simulation techniques can be used as a tool to visualize and analyze maintenance and inspection procedures, predict the time required for repairs, and develop a framework for maintenance activities that require remote handling. In ref. [11], a concept is introduced to assess the potential of inspection and maintenance processes in the aviation industry concerning the utilization of mixed reality systems. Four different scenarios are discussed, applying augmented or virtual reality devices in an industrial context. For the current case, the usability analysis on virtual reality devices is based on the development of a statistical analysis carried out based on sixty users (Table 2).

In order to not include learning or fatigue effects, the order to the tested devices was randomized. All the experiments are performed in a wide room with a background noise lower than 48 DB of SPL. For all the devices, the same workspace (room), the same distance from the floor (floor), and the same reference systems (to reduce lighting effects as well) have been set, while all the other parameters have been left by default with the factory ones.

To ensure the reliability and representativeness of the usability analysis, the participant sample was carefully balanced in terms of gender (30 males and 30 females) and age (ranging from 25 to 50 years). The selection process was conducted on a voluntary basis, targeting individuals with diverse backgrounds to reflect a realistic cross-section of potential industrial users. No formal tests of visual acuity or spatial aptitude were administered. All participants had normal or corrected-to-normal vision and none reported motor impairments that could interfere with task execution. Participants were not required to be VR experts or domain specialists. This choice reflects typical industrial conditions, where operators have heterogeneous prior exposure to VR. The use of generic, primitive-based tasks (pick, place, rotate, etc.) ensures that the evaluation does not depend on specific domain knowledge, and the weighted averaging of repeated trials mitigates the influence of individual outliers. Consequently, the comparative results are robust across different levels of user expertise. While participants were not selected based on specific professional profiles, all were sufficiently familiar with basic technological tools and provided a self-assessed level of VR experience using a 4-level scale (0 = no experience; 3 = high experience). This data, reported in Table 2, allowed us to ensure that the group included both novice and moderately experienced users, thus minimizing selection bias. The study involved non-invasive usability testing of virtual reality head-mounted displays with adult volunteers and did not include any clinical procedures, sensitive personal data, or vulnerable populations. According to institutional policies, formal ethical approval by an institutional review board or ethics committee was not required for this type of low-risk study. All methods were performed in accordance with the relevant guidelines and regulations. Participation was voluntary, and informed consent was obtained from all participants prior to their inclusion in the study.

For each user, 15 tasks were set up to be completed. Ten of these are defined as quantitative tasks, while the remaining five are defined as qualitative tasks. The users had to perform these tasks and provide quantitative and qualitative feedback for each device. The task order was kept constant across all participants to guarantee a standardized and identical learning curve, thus reducing inter-individual variability in the initial approach to the virtual environment. The tasks are designed to map directly to concrete industrial workflows, as summarized in Table 3. Primitive geometries are used intentionally to avoid confounding factors such as CAD model size or scene complexity, ensuring that performance differences are attributable to the headset and controllers rather than to rendering load. The benchmark is intended as a general, transparent tool for evaluating VR hardware usability; domain-specific scenarios (e.g., large CAD models, multi-user collaboration, live digital twin synchronization) are not included but can be integrated as extensions of this framework in future studies.

Although no formal spatial ability tests were conducted, the design of the tasks (especially those involving 3D manipulation, navigation, and spatial interaction) indirectly evaluated spatial reasoning and user adaptability. To reduce the influence of initial inexperience or fatigue, each task was performed in five repetitions per user, and a weighted average strategy was applied, assigning less significance to the first and last trials (weights of 0.1), as detailed in Section 3.3.

The feedback is based on some Key Performance Indicators (KPIs) [28] and on personal comments about the experiences. To ensure consistency in device handling, each participant was assisted by a trained technician responsible for verifying correct headset positioning and controller usage. The experiments were conducted in a controlled indoor environment, a laboratory space measuring 6 by 6 m, to ensure safe and repeatable movement conditions. Following data collection, the KPIs were subjected to inferential statistical analysis to assess whether performance differences between the VR headsets were statistically significant across tasks. For each task, a Repeated Measures Analysis of Variance (RM-ANOVA) was performed, treating the headset as a within-subjects factor [51]. The assumption of sphericity was tested using Mauchly’s test [52]; when violated (p<0.05), the degrees of freedom were adjusted using the Greenhouse–Geisser correction [53]. However, if the Greenhouse–Geisser epsilon (GG−ε) exceeded 0.75, the more liberal Huynh–Feldt correction was considered more appropriate [54]. Statistical significance was evaluated based on *p*-values with a threshold of α=0.05. To assess the practical relevance of the results, generalized eta-squared ($\eta_{G}^{2}$) values were computed, representing the proportion of variance in user performance attributable to the headset. Finally, Tukey’s Honest Significant Difference (HSD) [55] post hoc test was applied to determine which specific pairs of headsets showed significant differences.

All the tasks were implemented using the Unity3D 2022.3.10 engine (https://unity.com/, accessed on 12 March 2025) and the Unity XR Interaction Toolkit 2.5 (https://docs.unity3d.com/Packages/com.unity.xr.interaction.toolkit@2.5/manual/index.html, accessed on 12 March 2025), developing a single, platform-agnostic environment (the same scene to be executed on all the devices without modifications). The Unity physics engine (gravity, rigid body dynamics, and collision detection) was active for all tasks, not only for those explicitly involving throwing or catching (Tasks 8 and 10). Primitive geometric shapes and basic interactions were chosen instead of realistic CAD models or full digital twins to isolate low-level hardware responsiveness (controller buttons, tracking, visual latency) from confounding factors such as scene complexity or software optimization. This reductionist approach allows direct attribution of performance differences to the headset and controllers. Tests are performed on a high-performance workstation at the Virtual Prototyping Laboratory of the University of Rome Tor Vergata with the following characteristics: INTEL i9 12900KF CPU, 128 GB of RAM, Nvidia RTX 3070 8 GB. These specifications can support the development and implementation of VR-based experiences. This equipment is specifically chosen to reduce delays due to insufficient hardware, thus enhancing the reliability of the proposed method.

### 3.1. Quantitative Task Description

The quantitative tasks are so named because the KPIs are based on ANOVA evaluation criteria or quantitative KPIs defined ad hoc for each task. The ANOVA criterion is based on variance analysis; therefore, statistical methods are used to quantitatively measure the differences between the devices [56,57,58].

To methodologically isolate hardware performance from individual differences, such as varying visuospatial aptitudes, motor coordination, or spatial memory, a rigorous within-subject, repeated measures experimental design was deployed. Since every participant evaluated all five devices under a fully randomized presentation order, intrinsic user traits and cognitive profiles were effectively controlled across all hardware conditions, thus eliminating inter-subject confounding. Furthermore, by aggregating multiple trials per task and utilizing a large, demographically balanced sample (N=60), the protocol ensures high statistical power capable of distinguishing genuine hardware-driven performance indicators from user-dependent fluctuations.

The quantitative tasks cover aspects of kinematic manipulation (grab, pick, and place objects) [7], interactive real-time dynamics [8] and interface management [6]. The ten tasks are described in the following sections of this document.

#### 3.1.1. Task 1: Near-Field Manipulation—Pick and Place

**Rationale**: Assess the ability to pick and place objects at the user’s fingertips (reachable with the touch of controllers).

**Figure 1:**
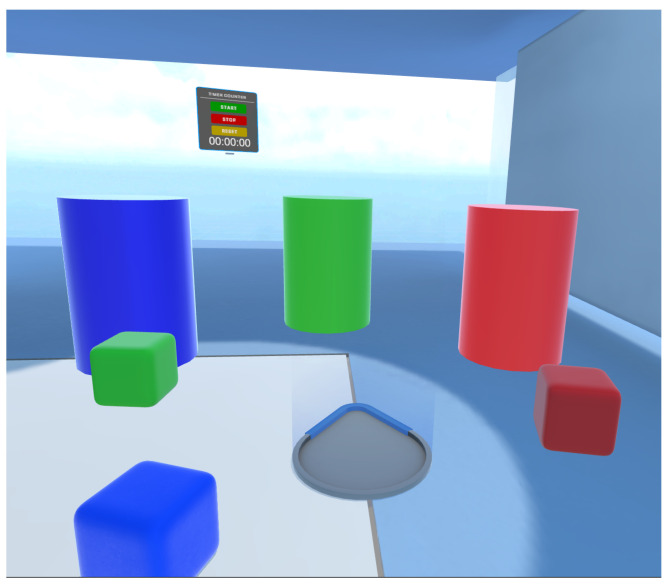
An example of the virtual environment of quantitative task 1.

**Description**: The user is asked to grab three cubes of different colors on a surface and place them on platforms with corresponding colors, placed at a distance of 2 m from the user (see Figure 1).

#### 3.1.2. Task 2: Far-Field Manipulation—Pick and Place

**Rationale**: Assess the ability to pick and place objects far from the user (reachable only with the controller’s ray).

**Figure 2:**
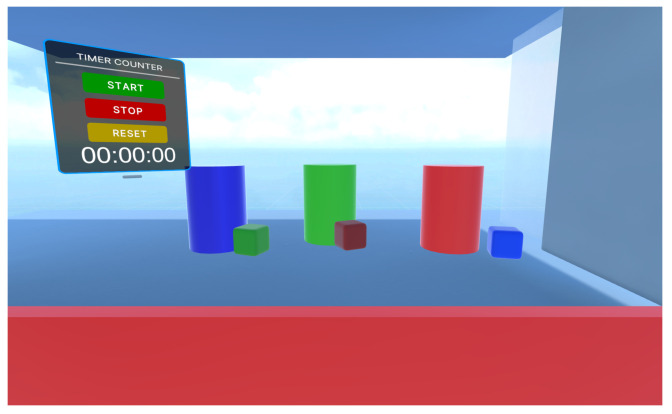
An example of the virtual environment of quantitative task 2.

**Description**: The user is asked to grab three cubes of different colors at a distance of 7 m, using the controller’s ray, and place them on platforms with corresponding colors, placed at 7 m from the user. A red barrier, placed at 1.5 m from the user, is included as a no-trespassing restrain (see Figure 2).

#### 3.1.3. Task 3: Near-Field Manipulation—Pick, Rotate and Insert

**Rationale**: Assess the ability to manipulate objects at the user’s fingertips (reachable with the touch of controllers).

**Figure 3:**
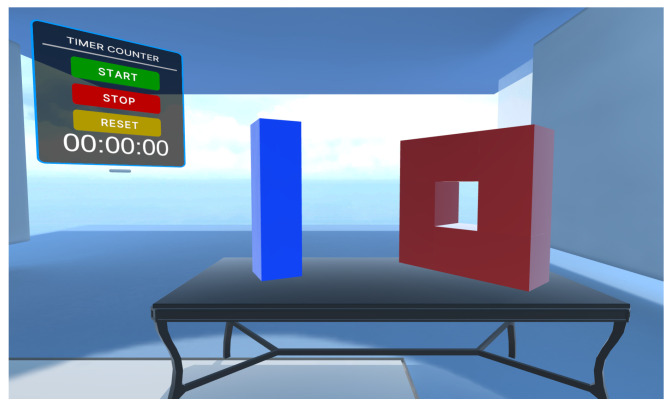
An example of the virtual environment of quantitative task 3.

**Description**: The user is asked to take one prismatic object on the outer surface, rotate it, and insert it into a cavity. The table, on which both the prismatic object and the cavity are positioned, is located 1 m away from the user (see Figure 3).

#### 3.1.4. Task 4: Far-Field Manipulation—Pick, Rotate and Insert

**Rationale**: Assess the ability to manipulate objects far from the user (reachable with the controller’s ray).

**Figure 4:**
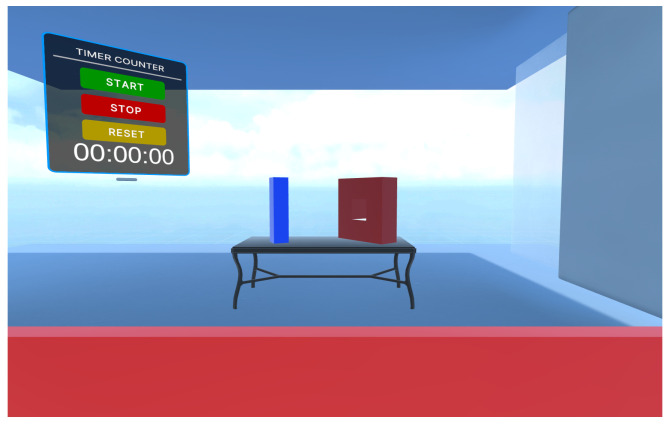
An example of the virtual environment of quantitative task 4.

**Description**: The user is asked to take one prismatic object, rotate it, and insert it into a cavity, using the controller’s ray. The table, on which the prismatic object and the cavity are positioned, is located 3.5 m away from the user. A red barrier, placed 1.5 m from the user, is employed as a no-trespassing restrain (see Figure 4).

#### 3.1.5. Task 5: Two-Hand Dynamics

**Rationale**: Assess how the user can interact with both hand controllers with objects subjected to physics.

**Figure 5:**
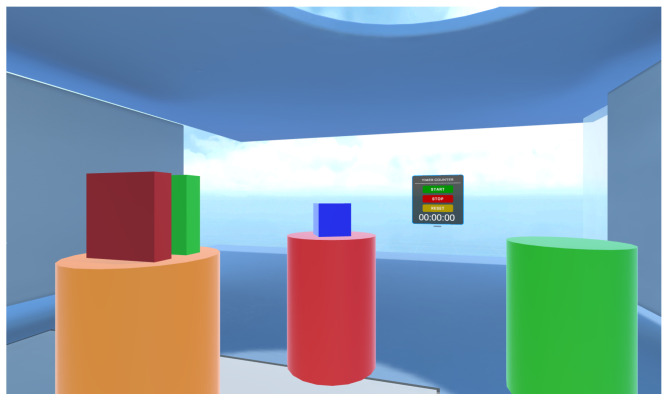
An example of the virtual environment of quantitative task 5.

**Description**: The user is asked to grasp two prismatic objects (one in each hand), placed on the user’s left side, to pick up with them a third object of prismatic shape, located in front of the user’s initial position, and move it onto another platform, placed on the user’s initial right side (see Figure 5).

#### 3.1.6. Task 6: Button Interaction

**Rationale**: Assess the interaction between the user and virtual buttons through multiple pressing.

**Figure 6:**
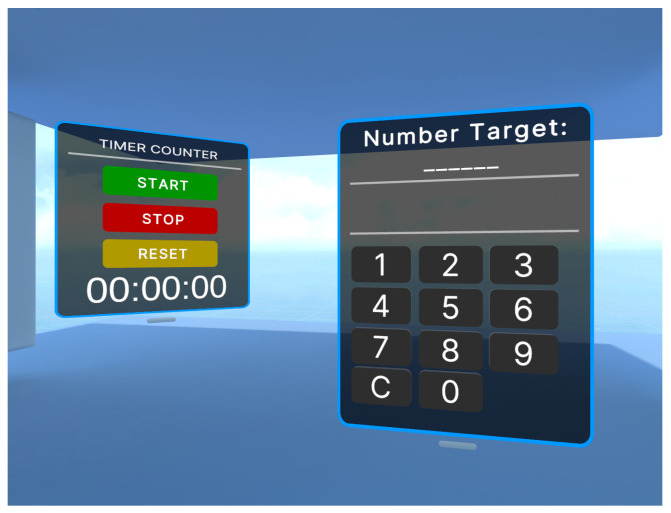
An example of the virtual environment of quantitative task 6.

**Description**: The user is asked to type a combination of six randomly generated numbers on a vertical numeric keypad placed at a distance of 1m (see Figure 6).

#### 3.1.7. Task 7: Teleporting

**Rationale**: Assess the ability in the recognition of teleporting and accuracy in pointing stations.

**Figure 7:**
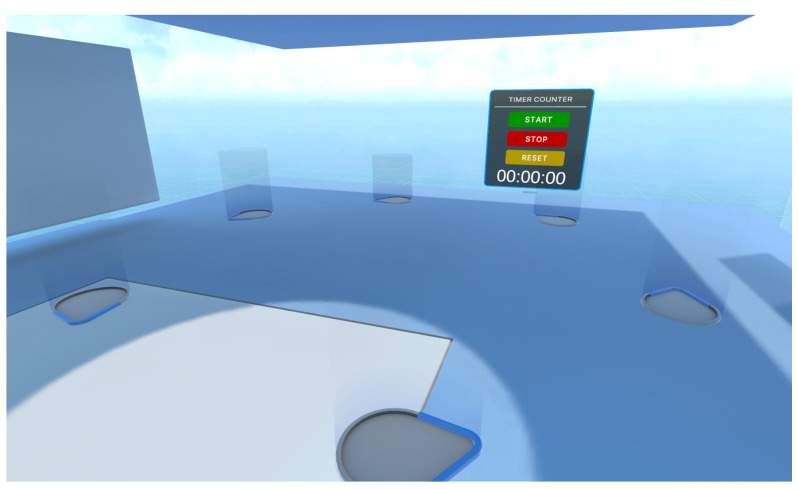
An example of the virtual environment of quantitative task 7.

**Description**: The user is asked to teleport in a sequence of six fixed stations placed at a relative distance of 3 m from each other (see Figure 7).

#### 3.1.8. Task 8: Dynamics—Pick and Throw

**Rationale**: Assess how the user can interact with objects subjected to physics and how the release action from the controller can influence the user’s perception of physical behavior.

**Figure 8:**
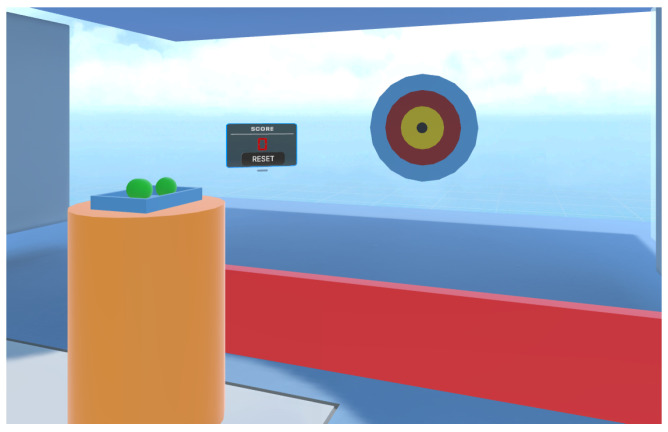
An example of the virtual environment of quantitative task 8.

**Description**: The user is asked to grab three spherical objects with a radius of 10 cm, placed at a distance of 1 m, and to throw them towards a vertical sticky dartboard, placed at a distance of 3.5 m. The dartboard has four different scoring zones: black scores 100 points, yellow scores 75 points, red scores 50 points, and blue scores 25 points. A red barrier, placed 1.5 m from the user, is employed as a no-trespassing restraint (see Figure 8).

#### 3.1.9. Task 9: Reading Canvas at Different Distances

**Rationale**: Assess the readability of text at different distances from the user.

**Figure 9:**
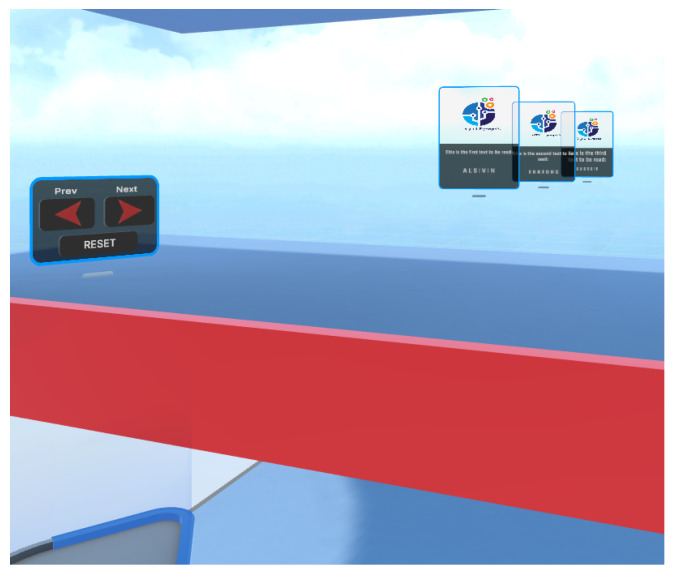
An example of the virtual environment of quantitative task 9.

**Description**: The user is asked to read 21 letters displayed on three different panels, with 7 letters on each one. The letters are written in uppercase using the Inter-Regular SDF font with a font size of 25 units. These panels are placed at three distances: 4.5 m, 6.5 m and 8.5 m. The user can navigate through the panels using the UI panel on the left. A red barrier, placed 1.5 m from the user, is employed as a no-trespassing restraint (see Figure 9).

#### 3.1.10. Task 10: Interactive Dynamics

**Rationale**: Assess how the user can interact with moving objects in space.

**Figure 10:**
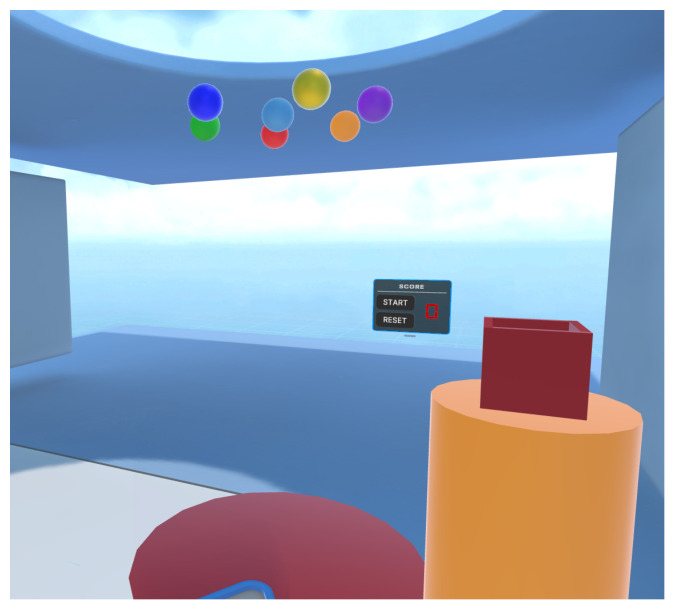
An example of the virtual environment of quantitative task 10.

**Description**: The user is asked to grab a hollow object and to catch five free-falling spheres from 2.5 m of height in a randomized sequence before they reach the ground. To increase the difficulty, seven spherical objects are present instead of five, challenging the user to catch the last sphere (see Figure 10).

### 3.2. Qualitative Tasks Description

For the qualitative tasks, a custom 5-point Likert scale [59,60] was adopted to collect subjective user judgments regarding comfort, visual clarity, and perceived interaction quality. The method consists of a series of quick-response questions to which users must assign a rating from 1 to 5, where 1 corresponds to “Strongly Disagree” and 5 to “Strongly Agree”. More details are provided in Section 3.4. The five qualitative tasks, from the eleventh to the fifteenth ones, are described below.

#### 3.2.1. Task 11: Hi-Res Model Inspection

**Rationale**: Assess the perception of the details of 3D models in the virtual environment.

**Figure 11:**
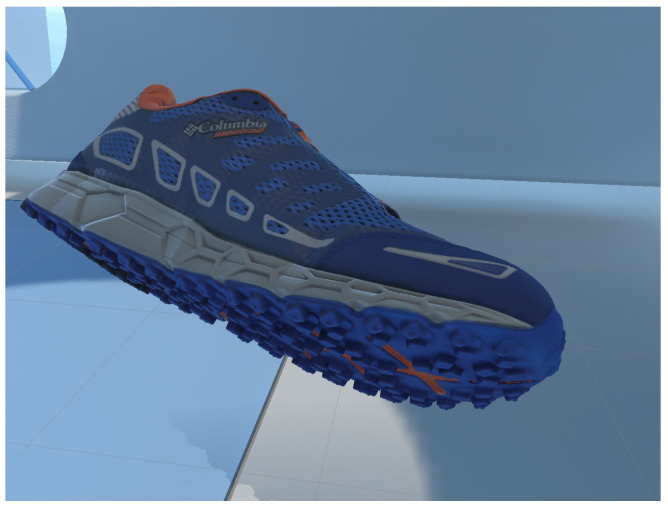
An example of the virtual environment of qualitative task 11.

**Description**: The user navigates a high-resolution texturized mesh model (100,000 triangles and 4K resolution texture), making a judgment on the sharpness of the details (see Figure 11).

#### 3.2.2. Task 12: Sound Listening

**Rationale**: Assess the perception of the sound and listening.

**Figure 12:**
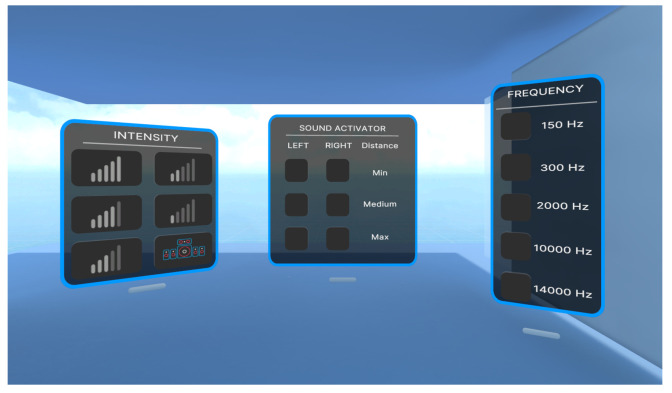
An example of the virtual environment of qualitative task 12.

**Description**: The user is asked to listen to a series of sounds and judge the clearness. There are three different panels located in front of the user: the left one is used to adjust the sound source intensity, the front panel allows the activation of the source coming from six different distances and location combinations and the right one allows the users to hear five different frequencies The sound sources are placed at 1 m, 5 m, and 20 m. The frequencies are 150 Hz, 300 Hz, 2 kHz, 10 kHz, and 15 kHz (see Figure 12).

#### 3.2.3. Task 13: Mid-Exposure Tolerability

**Rationale**: Assess the motion sickness and the sense of dizziness and alienation.

**Description**: The user is immersed in the virtual environment for 20 min and, at the end, expresses a tolerability judgment to the device. As explained by Chang et al. [36], 20 min is enough to perceive feelings of sickness.

#### 3.2.4. Task 14: Ergonomics and Comfort in Wearing

**Rationale**: Assess the ergonomics of head-mounted displays and controllers.

**Description**: The user is asked to judge the ergonomics of wearing the head-mounted displays and controllers assessing the weight, the screen quality, the lens, the buttons, and the adjustments.

#### 3.2.5. Task 15: Low-Light Environment Sensibility

**Rationale**: Assess the head-mounted display performance in low-light conditions.

**Figure 13:**
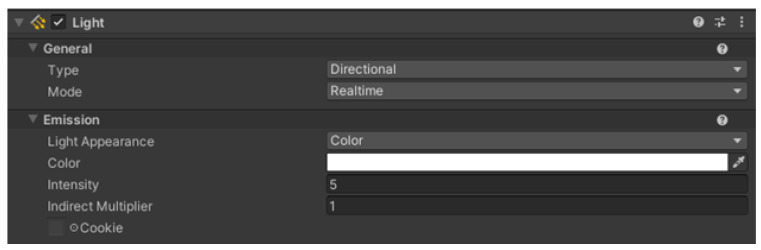
Properties of directional lights for the 5th level of intensity.

**Figure 14:**
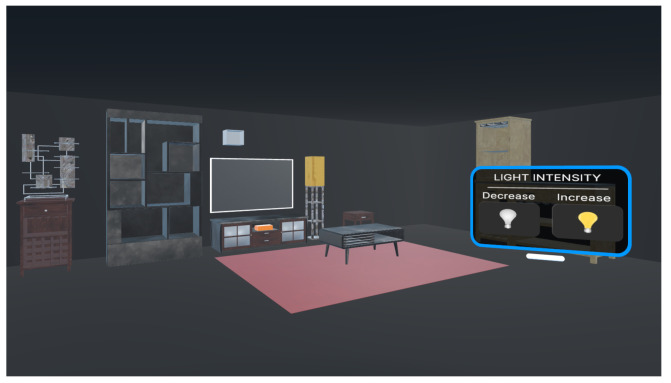
An example of the virtual environment of qualitative task 15.

**Description**: The user is asked to judge an ambient scene illuminated by four directional lights, positioned in the upper corners of the room, each with five different intensity levels ranging from 0 to 5 (Figure 13). The user can adjust the light intensities using a user interface (UI) panel. An overview of the virtual environment is shown in Figure 14.

### 3.3. Quantitative Task Assessment

For each quantitative task, to ensure significant results and eliminate erroneous data due to limited VR experience, each user is required to perform five trials. For each trial, a performance parameter (PP) is defined. To define a key PP (KPP), a weighted average of the PP obtained in each single trial is computed using the following formula [61]:

(1) $$KPP=\frac{\sum_{i=1}^{5}PP_{i}\cdot w_{i}}{\sum_{i=1}^{5}w_{i}}$$

where *i* is the trial, and $w_{i}$ is the corresponding weight. The definition of the weights is linked with the experience of the user in the development of the task. For this reason, the first and last trials are considered to have low significance due to users being inexperienced and overly experienced in task development. Following this rationale, the weights have been distributed as follows:

- Trials 1 and 5: the weight is 0.1;
- Trials 2 and 4: the weight is 0.25;
- Trial 3 the weight is 0.3.

For the first seven quantitative tasks, temporal KPIs have been defined. The users need to click a button to start the timer, perform the task, and then click a second button to stop the timer. In these cases, the PPs consist in the user’s time to complete the *i*-th trial. Once a reference time ($t_{ref}$) was defined as the mean value of the times for all users’ trials for the *j*-th task, and the KPP was calculated with Equation (1), each KPI was defined with the following formula:

(2) $$\left\{\begin{array}{cc} 1 & KPP_{j}<\frac{t_{ref}}{2}\\ KPI_{j}=\frac{3}{2}-\frac{KPP_{j}}{t_{ref}} & \frac{t_{r}ef}{2}\leq KPP_{j}\leq \frac{3}{2}t_{ref}\\ 0 & KPP_{j}>\frac{3}{2}t_{ref} \end{array}\right.$$

The threshold values were selected to map performance into an intuitive normalized scale, where completion within half the reference time was considered optimal and performance exceeding 150% of the reference time was considered unacceptable for industrial usage. According with this rationale, outlier behaviors were addressed within the KPI computation itself: for any performance time exceeding 150% of the task’s reference value, the KPI was automatically set to zero in accordance with Equation (2). This approach ensured that outliers did not affect the overall evaluation without requiring the exclusion of any participants.

**Figure 15:**
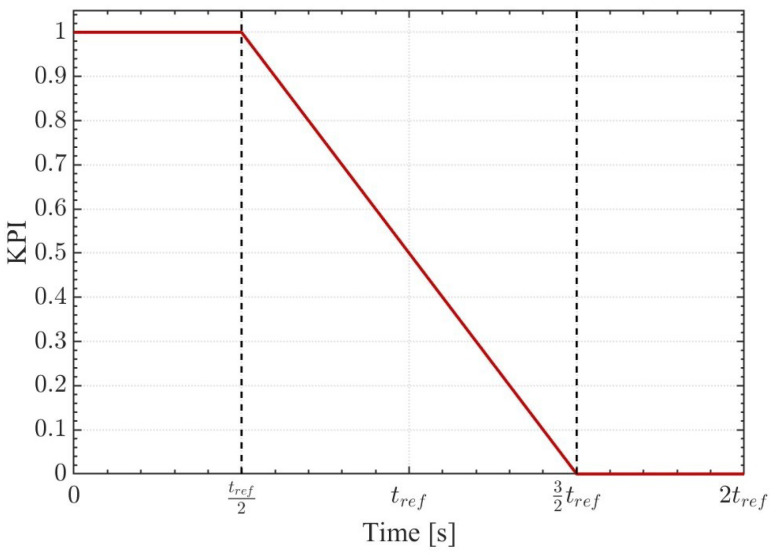
KPI assessment function for the first seven quantitative tasks.

Figure 15 shows the KPI assessment according to Equation (2). Note that a task is fully achieved with the maximum score when the time required for its completion is lower than the half of his reference values.

Reference values, i.e., $t_{ref}$, are computed as the average value among all the trials and all the users. Table 4 reports the computed valued of $t_{ref}$ for the set of sixty users that have tested the experiences.

For the last three quantitative tasks (tasks 8, 9, and 10), the PPs consist of the score of the respective task: in the eighth task, the PP is set equal to the target score result; for the ninth one, the PP is determined by the number of correct letters that users have read; meanwhile, for the tenth one, the PP is based on the number of balls the user has caught with the hollow box. The KPPs are equal to Equation (1). Meanwhile, the KPI is derived from the normalized results of the KPPs: Equations (3)–(5) represent the KPIs for the eighth (T8), ninth (T9), and tenth tasks (T10), respectively.

(3) $$KPI_{T8}=\frac{KPP_{T8}}{300}$$

(4) $$KPI_{T9}=\frac{KPP_{T9}}{21}$$

(5) $$KPI_{T10}=\frac{KPP_{T10}}{5}$$

### 3.4. Qualitative Task Assessment

In qualitative tasks, a 5-point Likert scale was used (1 = Very poor, 2 = Poor, 3 = Sufficient, 4 = Good, 5 = Very good). The user was asked to give a personal judgement for each task by selecting the corresponding numerical score. The normalized KPI was then computed as follows:

(6) $$KPI=\frac{score}{5}$$

This simple scale was preferred over comprehensive multi-item standardized questionnaires (such as the SUS, NASA-TLX, or SSQ) because each subjective task addressed a single, well-defined perceptual or ergonomic attribute (visual sharpness, sound clarity, motion sickness, ergonomics, low-light sensitivity) rather than broad, multidimensional latent constructs. From a methodological standpoint, multi-item scales were conceptually unnecessary and theoretically redundant for such atomistic evaluations [62,63]. Furthermore, introducing extensive multi-item questionnaires across our large-scale protocol (60 users, 5 devices, 15 tasks) would have forced each participant to evaluate thousands of distinct query inputs. In human–computer interaction workflows, such an extreme response burden induces severe user fatigue, leading to automated straight-lining and significantly compromising data integrity through response bias [64]. Because each subjective hardware attribute is measured via an independent, single-item metric, multi-item psychometric internal consistency indicators, such as Cronbach’s α, McDonald’s ω, or exploratory factor analysis (EFA), are mathematically inapplicable. Instead, measurement reliability and cross-study reproducibility are strictly ensured by explicit, discrete Likert anchors, identical environmental baselines, and a large sample size (N=60) [60], which is fully sufficient for the benchmarking purposes of this study.

## 4. Usability Analysis Results

The data collected during the tests offer an enlightening perspective on the devices involved in the usability analysis, highlighting their strengths and weaknesses through various tasks. Numerical results from the tests conducted on 60 users are summarized in the following figures (Figure 16, Figure 17, Figure 18, Figure 19, Figure 20, Figure 21, Figure 22, Figure 23, Figure 24, Figure 25, Figure 26, Figure 27, Figure 28, Figure 29 and Figure 30), showing, for each device, the mean value and standard deviation of users’ KPIs for the corresponding task.

Furthermore, the KPIs were used as dependent variables in a repeated measures ANOVA to assess the statistical significance of the differences observed across tasks and devices. Table 5 reports the results of Mauchly’s test for each task, which was conducted to verify the assumption of sphericity.

For all tasks except Task 1, the *p*-values are below the conventional threshold of 0.05, indicating that the sphericity assumption is violated. Therefore, correction methods were applied to adjust the degrees of freedom accordingly. Specifically, for Tasks 2, 3, 6, 9, and 13, the Greenhouse–Geisser correction was used, as the GG-ε was below the threshold of 0.75. For Tasks 4, 5, 7, 8, 10, 11, 12, 14, and 15, the Huynh–Feldt correction was applied, since the GG-ε exceeded the 0.75 threshold. Once the appropriate corrections were applied, the *p*-values and generalized $\eta^{2}$ were calculated for each task (Table 6).

All *p*-values are below the threshold of 0.05, indicating that the effects observed for all tasks are statistically significant. The generalized $\eta^{2}$ values ($\eta_{G}^{2}$) provide an indication of effect size, that is, how much of the variance in the dependent variable (KPIs) can be attributed to the headset. More specifically, high $\eta_{G}^{2}$ values suggest that the choice of device plays a considerable role in performance for that task, whereas low values suggest that the device has a smaller impact. For instance, Tasks 4 ($\eta_{G}^{2}=0.456$), 6 ($\eta_{G}^{2}=0.603$), 10 ($\eta_{G}^{2}=0.452$), 13 ($\eta_{G}^{2}=0.406$) and 14 ($\eta_{G}^{2}=0.467$) exhibit large effect sizes. This indicates that, for these tasks, performance varies substantially depending on the headset used, making these tasks particularly informative. Finally, the statistical procedure was completed with a post hoc analysis using Tukey’s test. While the 15 tasks assess independent usability dimensions (meaning a cross-task alpha correction is not strictly mandatory under independent operational constructs), we formally verified the mathematical robustness of our main effects against Type I error inflation. For all 15 primary RM-ANOVAs, the original omnibus F-tests yielded an extremely high separation with p<0.001. Consequently, even when applying the most conservative Bonferroni adjustment across all 15 tasks—which lowers the significance threshold to $\alpha_{adj}=0.05/15=0.0033$—all 15 main macro-effects remain highly statistically significant (p<0.001), leaving the scientific conclusions of the benchmark completely unaltered. Furthermore, a sensitivity check on the inner pairwise comparisons confirms that switching from Tukey’s HSD to a post hoc Bonferroni adjustment yields identical results: highly significant pairs consistently retain p<0.001, while non-significant pairs remain at p=1.000. Within each individual task, the FWER for the 10 pairwise comparisons was strictly controlled using Tukey’s HSD post hoc test. By considering the list of VR devices and their acronyms as reported in Table 7.

The results of the Tukey’s HDS post hoc test are reported from Table 8, Table 9, Table 10, Table 11, Table 12, Table 13, Table 14, Table 15, Table 16, Table 17, Table 18, Table 19, Table 20, Table 21 and Table 22.

**Figure 16:**
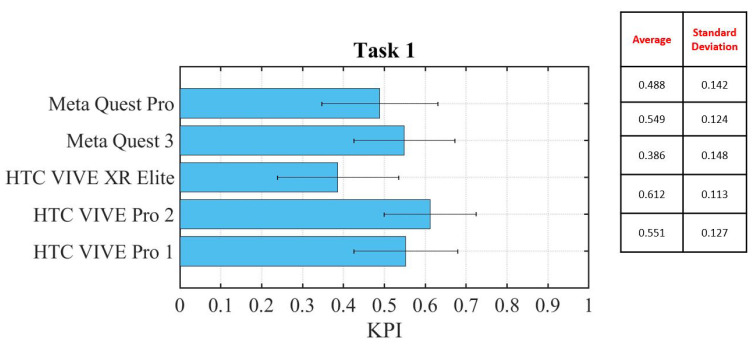
Mean KPI and standard deviation for Task 1 (near-field manipulation—pick and place).

**Figure 17:**
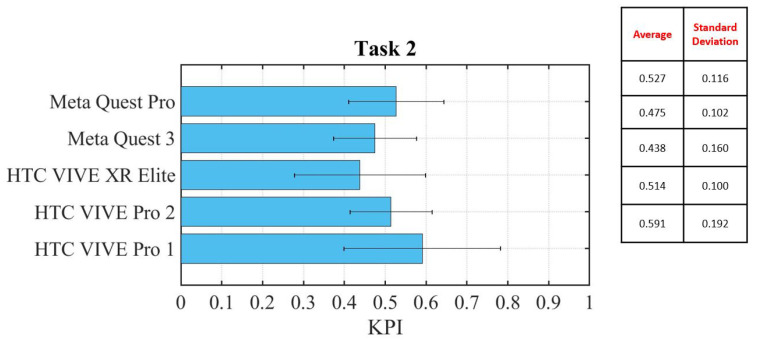
Mean KPI and standard deviation for Task 2 (far-field manipulation—pick and place).

**Figure 18:**
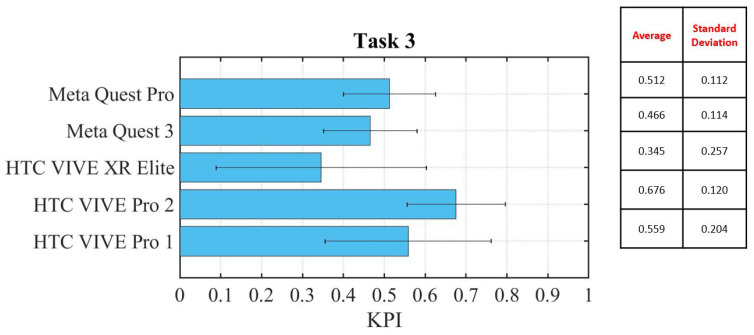
Mean KPI and standard deviation for Task 3 (near-field manipulation—pick, rotate and insert).

**Figure 19:**
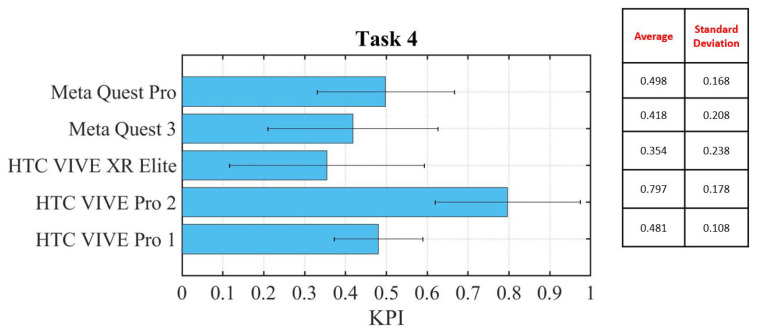
Mean KPI and standard deviation for Task 4 (far-field manipulation—pick, rotate and insert).

**Figure 20:**
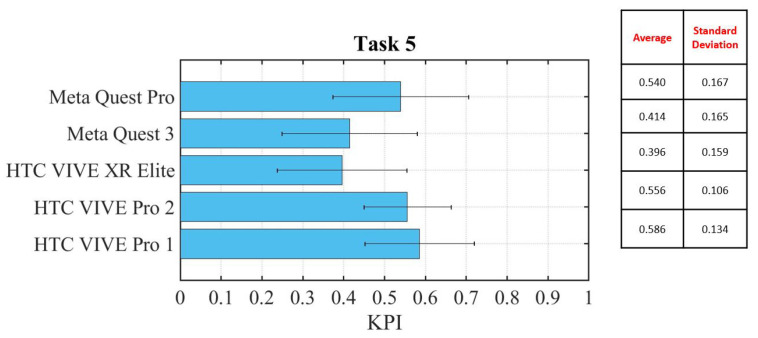
Mean KPI and standard deviation for Task 5 (two-hand dynamics).

In addition to numerical results, user comments and considerations have been emphasized. From Figure 16, Figure 18 and Figure 20, it can be observed how the HTC VIVE Pro 2 device, followed by the two Meta headsets and the HTC VIVE Pro 1, seems to facilitate close-range object manipulation and handling. The manipulation of the objects occurred with the trigger button. Despite the controllers of Meta and HTC VIVE XR Elite appearing more ergonomic and lighter compared to those of Pro 1 and Pro 2 devices, the majority of users found greater practicality in the use of the latter, particularly due to more consistent input feedback. Furthermore, the controllers of Meta devices are lighter than those of the XR Elite. However, users noticed in the Meta devices, particularly in the Meta Quest 3, an impulsive release of objects, producing an unexpected bounce of the manipulated object upon release. These bounces consist of an initial velocity of the object not congruent with that of the user’s hand, altering the subsequent physical simulation. From Table 8, Table 10 and Table 12, with the support of Table 7, it is possible to identify the statistically significant comparisons between headsets (*p*-value < 0.05). The HTC VIVE XR Elite proves to be statistically less suitable for performing close-range object manipulation tasks compared to the other devices analyzed. Conversely, the HTC VIVE Pro 2 appears, on average, to be the most statistically suitable headset. A marked improvement in the release physics of objects was observed in the transition from the HTC VIVE Pro 1 to the Pro 2. Figure 17 and Figure 19 and Table 9 and Table 11 show the tasks related to handling and movement of objects far from the user, where manipulation occurs through pointing rays originating from the controllers. According to the test results, especially about task 4, the rays cast by the HTC VIVE Pro 2 were more accurate and stable than those from the other devices, facilitating more accurate movements. This supports the observation that Tasks 3 and 4 (Figure 18 and Figure 19),which involve high-precision assembly operations, exhibited a clear performance advantage of the VIVE Pro 2 over the XR Elite, confirming its suitability for demanding manipulation tasks. In Task 5 (Figures Table 12 and Figure 20), which requires the two controllers to operate synchronously, the HTC VIVE Pro and Meta Quest Pro systems outperformed the other devices. This result can be attributed to the fact that the HTC VIVE Pro relies on an outside-in tracking approach based on external base stations, which is generally associated with high tracking accuracy and robustness. Remarkably, the Meta Quest Pro achieved comparable performance despite employing an inside-out tracking system. This can be explained by the advanced self-tracking capabilities of the Touch Pro controllers, which integrate onboard cameras and dedicated processing to improve controller localization and reduce tracking errors during tasks requiring precise controller synchronization.

**Figure 21:**
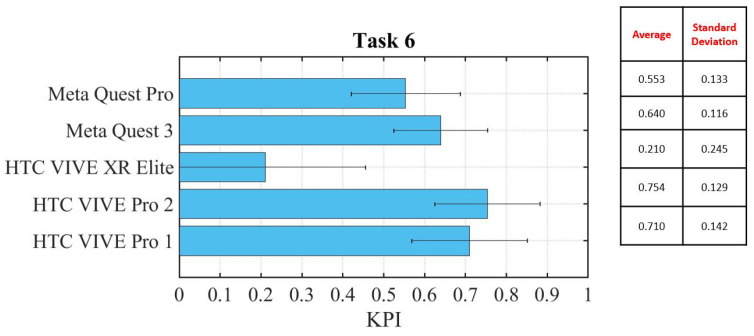
Mean KPI and standard deviation for Task 6 (button interaction).

**Figure 22:**
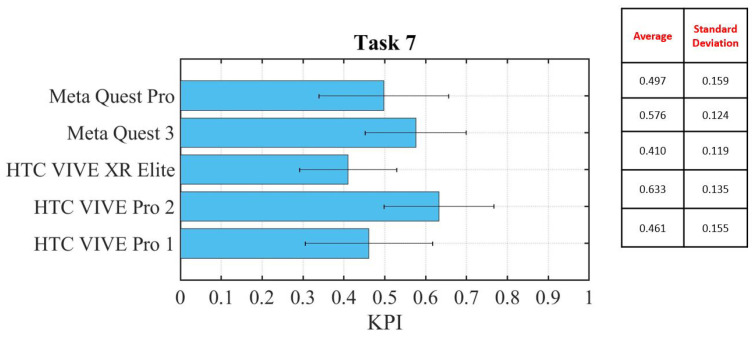
Mean KPI and standard deviation for Task 7 (teleporting).

**Figure 23:**
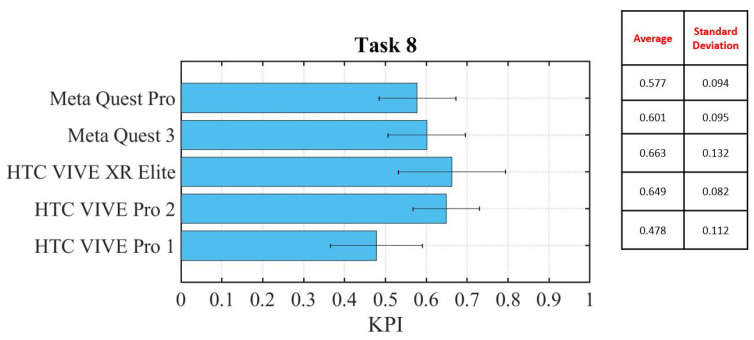
Mean KPI and standard deviation for Task 8 (dynamics—pick and throw).

**Figure 24:**
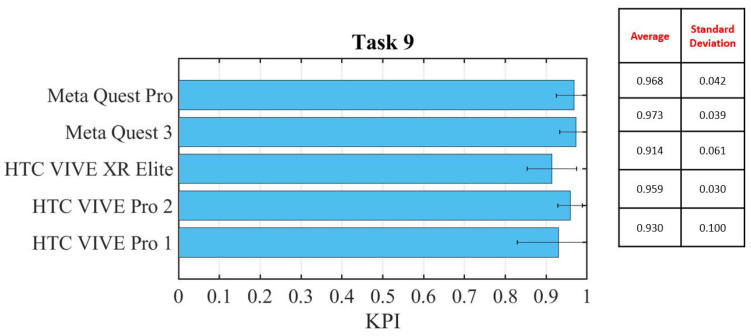
Mean KPI and standard deviation for Task 9 (reading canvas at different distances).

**Figure 25:**
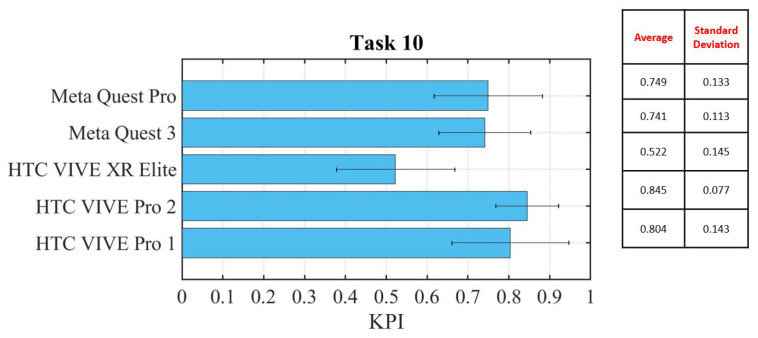
Mean KPI and standard deviation for Task 10 (interactive dynamics).

Regarding the grip button, the 6th task (Figure 21, Table 13) reveals a significant performance gap between the HTC VIVE XR Elite and the other devices. An issue was identified concerning unintended double inputs from the grip button, often leading to errors when entering the six-number combination and causing prolonged task durations. This corroborates the decline in performance observed in Task 6 (Figure 21), where users directly attributed delays to these interaction problems. Task 7 involves a teleportation sequence using the thumbstick for Meta devices and the HTC VIVE XR Elite, and the trackpad button for the HTC VIVE Pro 1 and 2 (Figure 22, Table 14). Users preferred the trackpad command for its faster recognition and execution, though they reported that the touch-based interaction could occasionally lead to unintended activations. This aligns with the findings of limited performance differences in teleport-based locomotion tasks (Task 7), where only marginal gaps were observed across headsets. Task 8 (Figure 23, Table 15) offered deeper insights into the release dynamics: despite the HTC VIVE XR Elite achieving higher scores in trajectory linearity due to its external tracking system, users perceived greater physical realism in the object behavior from the HTC VIVE Pro 1 and 2, suggesting that simulation fidelity is not solely dictated by tracking accuracy but also by integration with the physics engine. The 9th task (Figure 24, Table 16) shows the results related to visual quality. All devices offered excellent rendering for the nearest panels, but a difference was observed on the third distant panel. Based on both objective data and subjective feedback, the Meta Quest 3 and the Meta Quest Pro delivered the most enjoyable visual quality, followed by the HTC VIVE Pro 2. However the *p*-values between HTC VIVE Pro 2 and two Meta devices are higher than 0.05, so this could be not statistically determined. These results align with the low variance reported in Task 9 (Figure 24), confirming the robustness of visual performance. The blurriness perceived at the top of the field of view for the HTC VIVE Pro 1 is likely linked to its optical system, while the XR Elite showed a less comfortable visual experience despite its resolution, likely due to design limitations. Task 10 (Figure 25, Table 17) evaluated spatial perception. The XR Elite was rated as providing a suboptimal perception experience: during the ball-falling sequence, users were required to quickly move their heads to follow the motion, and some reported perceiving a slight elliptical distortion of the vertical space. These findings are consistent with those of Task 10 (Figure 25), where the XR Elite ranked lower than the VIVE Pro 2, VIVE Pro 1, and Meta Quest Pro. In contrast, the Meta Quest 3 was reported to offer smoother scene transitions and improved visual comfort, enhancing spatial awareness.

### 4.1. Qualitative Task Results and Discussions

**Figure 26:**
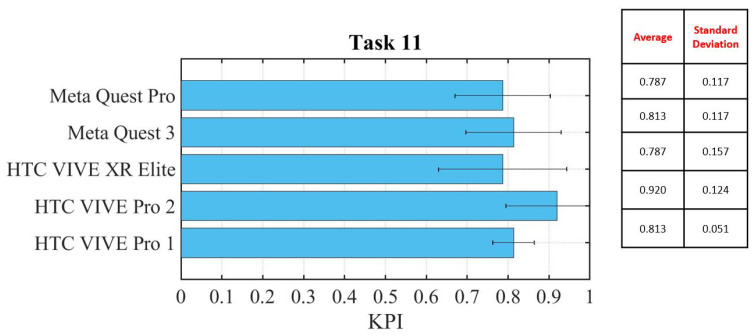
Mean KPI and standard deviation for Task 11 (high-resolution model inspection).

**Figure 27:**
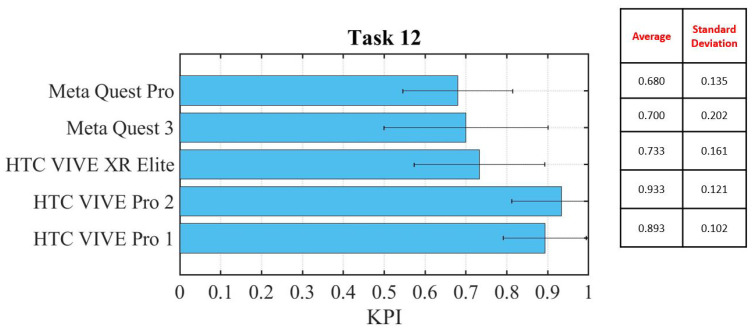
Mean KPI and standard deviation for Task 12 (sound listening).

**Figure 28:**
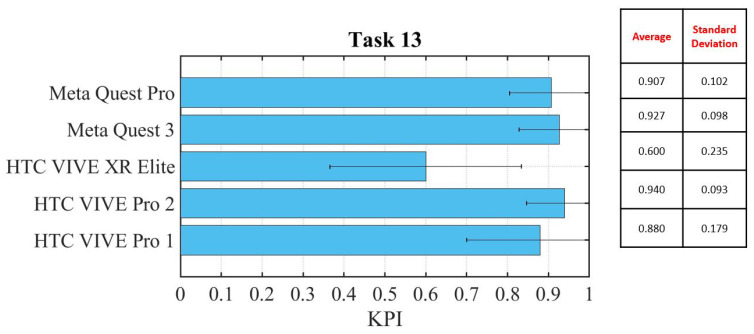
Mean KPI and standard deviation for Task 13 (mid-exposure tolerability).

**Figure 29:**
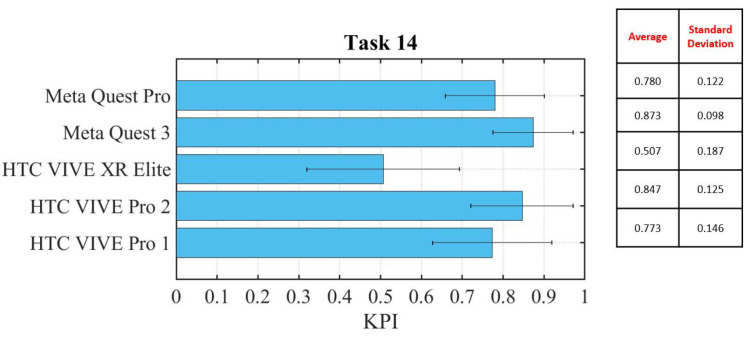
Mean KPI and standard deviation for Task 14 (ergonomics and comfort in wearing).

**Figure 30:**
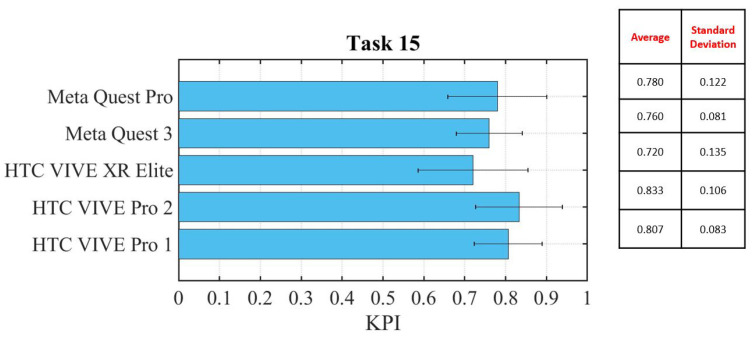
Mean KPI and standard deviation for Task 15 (low-light environment sensibility).

The qualitative assessment from Task 11 (Figure 26, Table 18) confirmed a clear user preference for the HTC VIVE Pro 2 in high-resolution inspection tasks, supported by a relatively low data dispersion. This observation reinforces its utility in precision-dependent virtual assembly environments. As for the devices’ weight and comfort (Task 12, Figure 27, Table 19), all users consistently reported a clear advantage for the Meta Quest Pro and Meta Quest 3, followed by the XR Elite. The HTC VIVE Pro 1 and 2 were perceived as significantly heavier and more cumbersome, especially during longer sessions. This perception matches the increase in reported fatigue and discomfort over time observed during the experimental protocol. Despite the visual superiority of the VIVE Pro 2 in high-resolution tasks, its headset structure appears to compromise extended usability, which may limit its adoption in immersive industrial simulations that require prolonged engagement. In Task 13 (Figure 28, Table 20), which evaluated interaction with the surrounding environment and the ability to identify scene boundaries, the Meta Quest Pro and Quest 3 demonstrated excellent performance, thanks to the integrated passthrough system and the headset’s ergonomics, which facilitated quick and natural physical orientation. The XR Elite, despite having a passthrough mode, showed limitations due to its reduced field of view and slight latency in scene rendering, especially during quick head movements. This was consistent with the subjective reports of users, who expressed a preference for Meta devices in terms of contextual awareness and safety during transitions between virtual and physical environments. Task 14 (Figure 29, Table 21) concerned the usability and precision of menu interaction using ray pointers. The results showed a strong performance by the HTC VIVE Pro 2 and the Meta Quest Pro, confirming their superior pointing stability observed earlier in Tasks 2 and 4. In contrast, the XR Elite exhibited occasional jitter and pointer drift, particularly when menus were positioned toward the edges of the user’s field of view. This diminished accuracy was reflected in the slower completion times and increased user frustration reported during the test. In the final task, Task 15 (Figure 30, Table 22), which involved the use of the thumbstick or trackpad for interacting with a virtual control panel, the difference in device performance was less pronounced. However, some users noted that the trackpad on the HTC VIVE Pro 1 and 2 offered more accurate feedback compared to the thumbsticks of the Meta devices, especially when fine directional input was needed. Nonetheless, the overall ratings suggest that both input systems were acceptable for such interactions, with preferences largely influenced by familiarity and prior VR experience.

### 4.2. Integrated Discussion of Performance Patterns

**Figure 31:**
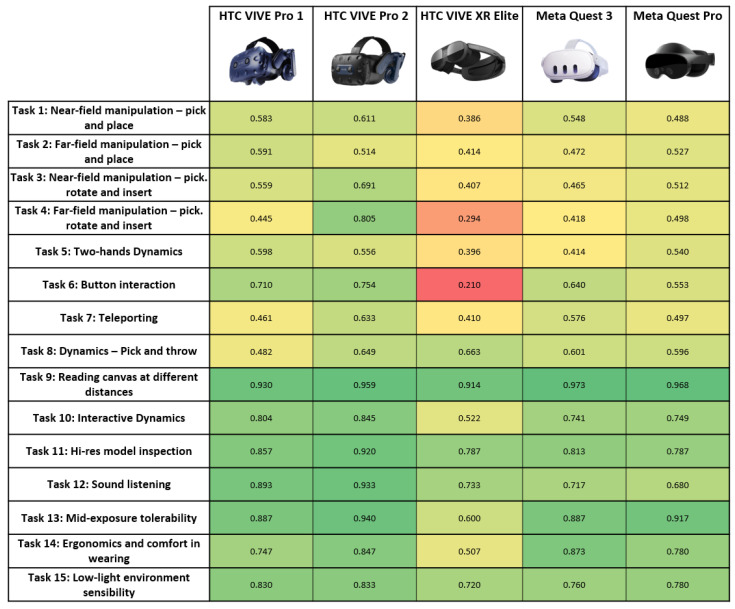
KPI mean values for each device and each task.

As shown by the results, as an overall assessment, the HTC VIVE Pro 2 device appears to be the most practical for virtual assembly applications, thanks to the ergonomics of the trigger button, due to excellent spatial perception and accurate tracking and manipulation of close and far objects. It seems that the HTC VIVE Pro 1 and Meta Quest Pro headsets yield good results regarding object manipulation; slightly less so for the Meta Quest 3 and HTC VIVE XR Elite. Concerning the grip button (side button), the HTC VIVE XR Elite seems to have some issues with releasing the button. Headsets equipped with thumbsticks (HTC VIVE XR Elite, Meta Quest 3, and Meta Quest Pro) appear to facilitate users in controlling input commands in terms of stability, while headsets with trackpads (HTC VIVE Pro 1 and 2) seem to speed up execution, risking a loss in control due to unwanted touch input. Regarding visual tasks, it appears that Meta headsets and the HTC VIVE Pro 2 favor the execution of a virtual inspection of a system, while the HTC VIVE XR Elite seems to have a slightly lower score. The VIVE PRO devices can be used in applications where audio feedback is relevant, in contrast to the Meta Quest 3. However, the Meta Quest Pro, in particular, does not appear the best choice for applications with high-frequency sounds. For long-term use, the Meta Quest Pro and Meta Quest 3 seem to have the best trade-off between physical and visual comfort. The physical comfort and sense of ease with the HTC VIVE XR Elite are very subject-dependent: users with experience in virtual reality and without glasses may find this headset suitable for long-term use, but new users found it less comfortable. The HTC VIVE Pro 1 and 2 devices are suitable for a limited exposure time due to the weight of the headsets. The mean values of the devices’ KPI for each task are reorganized in Figure 31. Despite having highlighted the average values of the KPIs, it is necessary to take into consideration the wide range of the standard deviation. This means that, despite the effort made to analyze the objective, some factors necessarily depend on the user.

## 5. Conclusions

This study proposed a comprehensive methodology to evaluate the usability of virtual reality headsets for industrial applications with hand-held controllers. Five commercially available devices were tested by sixty participants (30 male and 30 female, aged 25–50), balanced by demographics and prior VR experience, who performed fifteen tasks representative of typical industrial operations, including object manipulation, assembly validation, surface inspection, and auditory anomaly detection. Devices were assessed through objective metrics (task completion time, accuracy) and subjective feedback (comfort, ease of use, interaction quality, visual feedback).

The use of primitive, repeatable tasks allows the isolation of fundamental interaction primitives (pick, place, rotate, throw) that underlie complex industrial operations. This controlled approach enables direct comparison of device tracking, latency, and ergonomics without confounding variables such as scene complexity or CAD model size. Given the lack of prior comparative data on these specific enterprise-grade devices under standardized workflows, this framework deliberately adopts an exploratory, hypothesis-generating approach. Rather than testing rigid a priori assumptions, the methodology establishes a comprehensive empirical baseline from which clear, directional hypotheses can now be formulated for future confirmatory studies.

Inferential statistical analysis has adopted to evaluate the significance of observed differences across devices and participants, and to ensure the robustness and generalizability of the findings. The statistical analysis (e.g., ANOVA, post hoc tests) confirmed that significant differences exist among devices in terms of performance and user satisfaction. However, the presence of high standard deviation values, especially in subjective responses, indicates the persistence of strong user-dependent factors, such as physical characteristics (e.g., interpupillary distance, head shape), sensory perception, and prior experience. The results showed that some headsets are preferable for precision tasks requiring accurate tracking, while others are better suited for visual clarity, comfort, or prolonged sessions. No single device proved superior across all dimensions, emphasizing that hardware selection must be tailored to task requirements and user needs.

In conclusion, the proposed methodology combines technical benchmarking with rigorous usability testing and inferential statistical validation, offering a practical framework for evaluating VR systems in industrial contexts. While the reliance on primitive geometries ensures strict internal validity by eliminating software-rendering bottlenecks, it represents a trade-off that limits immediate ecological generalization to multi-million polygon scenes. Nonetheless, the benchmark is structurally modular; future work will extend this baseline layer by substituting geometric primitives with domain-specific industrial CAD models and dimensional tolerances, while retaining the validated KPI structures and statistical framework. Additionally, future developments will explore mixed reality platforms, long-term training scenarios, and direct hand interaction without controllers.

## Figures and Tables

**Table 1:** Technical specifications of the devices under investigation.

	HTC Vive Pro 1	HTC Vive Pro 2	Meta Quest Pro	HTC Vive XR Elite	Meta Quest 3
	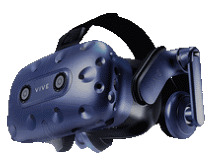	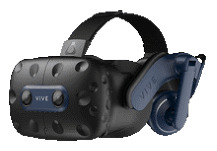	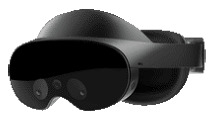	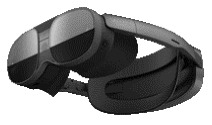	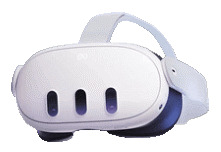
General Info					
Manufacturer	HTC	HTC	Meta	HTC	Meta
Device Type	PC-Powered VR	PC-Powered VR	Standalone VR	Standalone VR	Standalone VR
Platform	SteamVR, Viveport	SteamVR, Viveport	Meta Quest	Viveport	Meta Quest
Release Date	4 April 2018	3 June 2021	25 October 2022	31 March 2023	10 October 2023
Optics					
Optics		Dual-Element Fresnel Lenses	Pancake Lenses	Pancake Lenses	Pancake Lenses
IPD Range	61–72 Mm Hardware Adjustable (manual)	57–70 Mm Hardware Adjustable (manual)	55–75 Mm Hardware Adjustable (manual)	54–73 Mm Hardware Adjustable (manual)	58–71 Mm Hardware Adjustable (manual)
Passthrough	Dual Passthrough Cameras	Dual Passthrough Cameras	Color Passthrough	16MP RGB Camera	Dual 10 PPD Color Passthrough Cameras
Display					
Display Type	2 X AMOLED	2 X LCD Binocular	2 X LCD Binocular	2 X LCD Binocular	2 X LCD Binocular
			Local Dimming		
Resolution	1440 × 1600 Per-Eye	2448 × 2448 Per-Eye	1800 × 1920 Per-Eye	1920 × 1920 Per-Eye	2064 × 2208 Per-Eye
Refresh Rate	90 Hz	120 Hz	90 Hz	90 Hz	120 Hz
			72 Hz Mode Available		
Subpixel Layout	PenTile Diamond	RGB Stripe	RGB Stripe	RGB Stripe	RGB Stripe
	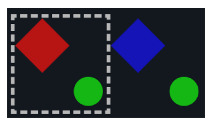	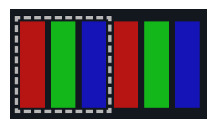	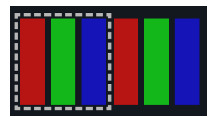	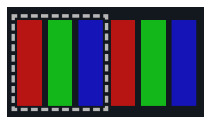	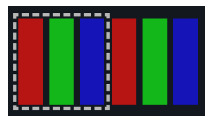
	2 Subpixels Per Pixel	3 Subpixels Per Pixel	3 Subpixels Per Pixel	3 Subpixels Per Pixel	3 Subpixels Per Pixel
Image					
Visible FoV	98° Horizontal	116° Horizontal	106° Horizontal	110° Diagonal	110° Horizontal
	98° Vertical	96° Vertical	95.57° Diagonal		96° Vertical
		113° Diagonal			
Rendered FoV	107.06° Horizontal	116.52° Horizontal	108° Horizontal	102.13° Horizontal	
	107.71° Vertical	96.49° Vertical	95.57° Vertical	91.27° Vertical	
	110.48° Diagonal	113.3° Diagonal	111.24° Diagonal	116.04° Diagonal	
Binocular Overlap	90.46°	79.83°	79.72°	80.93°	
Average Pixel Density	14.58 PPD Horizontal	24.93 PPD Horizontal	19.17 PPD Horizontal	20.97 PPD Horizontal	
	13.36 PPD Vertical	25.37 PPD Vertical	18.83 PPD Vertical	21.03 PPD Vertical	
Foveated Rendering	X	X	V	X	X
Device Embodiment					
Dimensions					
Weight	550 G Without Headstrap	850 G With Headstrap	722 G With Headstrap	625 G With Headstrap	515 G With Headstrap
	800 G With Headstrap			“With” “Battery Cradle” “Headstrap. With Glasses-Style Arms: 273 G”	
Material	Plastic, Foam Facial Interface	Plastic, Foam Facial Interface	Plastic, Foam Facial Interface	Plastic, Foam Facial Interface	Plastic, Foam Facial Interface
Headstrap	Hard Padded Retractable Strap	Hard Padded Retractable Strap	Hard Padded Retractable Strap	Hard Padded Retractable Strap	Flexible Fabric Strap
				Replaceable Headstrap With Hot-Swappable Battery	
Tracking					
Tracking Type	6 DoF Marker-Based	6 DoF Marker-Based	6 DoF Inside-Out Via 5 Integrated Cameras	6 DoF Inside-Out Via 4 Integrated Cameras	6 DoF Inside-Out Via 4 Integrated Cameras
				Also Includes Depth Sensor	Also Includes Depth Sensor
Tracking Frequency	1000 Hz	1000 Hz	X	X	X
	Rotational Tracking Frequency. Positional Frequency: 100 Hz	Rotational Tracking Frequency. Positional Frequency: 100 Hz			
Base Stations	2 X Vive Base Station	2 X SteamVR 2.0	X	X	X
Eye Tracking	X	X	V	X	X
Face Tracking	X	X	V	X	X
Hand Tracking	X	X	V	V	V
Body Tracking	X	X	X	X	X
Controllers					
Controllers	2 X Vive Pro Controller 6 DoF	2 X Vive Pro Controller 6 DoF	2 X Meta Quest Touch Pro Controller 6 DoF	2 X Vive XR Controller 6 DoF	2 X Meta Quest Touch Plus Controller 6 DoF
Weight	203 G	203 G	164 G	142 G	515 G
Input Methods	Trackpad, Face Buttons, Index Trigger, Grip Buttons	Trackpad, Face Buttons, Index Trigger, Grip Buttons	Capacitive Face Buttons, Capacitive Joystick, Capacitive Touch Pad, Capacitive Index Trigger, Middle Finger Trigger, Removable Stylus Attachment	Capacitive Face Buttons, Capacitive Joystick, Capacitive Touch Pad, Capacitive Index Trigger, Middle Finger Trigger	Capacitive Face Buttons, Capacitive Joystick, Capacitive Touch Pad, Capacitive Index Trigger, Middle Finger Trigger
Finger Tracking	Partial Thumb And Index Finger Tracking	Partial Thumb And Index Finger Tracking	Partial Finger And Thumb Tracking Via Capacitive Sensors	Partial Finger And Thumb Tracking Via Capacitive Sensors	Partial Finger And Thumb Tracking Via Capacitive Sensors
Haptics	V	V	V	V	V
Batteries	Rechargeable 6 h Battery Life	Rechargeable 6 h Battery Life	Rechargeable 8 h Battery Life	Rechargeable 15 h Battery Life	AA
Sound					
Speakers	Integrated Stereo Headphones	Removable Stereo Headphones	Integrated Stereo Speakers	Integrated Stereo Speakers	Integrated Stereo Speakers
Microphone	V	V	V	V	V
3.5 mm Audio Jack	X	V	V	X	V
Connectivity					
Ports	X	USB-C	USB Type-C, Charging Contacts	2 X USB 3.2 Gen 1 Type-C	USB Type-C, Charging Contacts
Wired Video	HDMI, USB-C 3.0	DisplayPort 1.2, USB 3.0	USB Type-C	USB-C	USB Type-C
			Oculus Link		Oculus Link
Wireless Video	X	Available Via VIVE Wireless Adapter, Sold Separately	WiFi Streaming	WiFI Streaming	WiFi Streaming
			Virtual Desktop, AirLink		Virtual Desktop, AirLink
WiFi	X	X	WiFi 6E	WiFi 6E	WiFi 6E
Bluetooth	X	Bluetooth	Bluetooth 5.2	Bluetooth 5.2 LE	Bluetooth 5.2
System					
Chipset			Qualcomm Snapdragon XR2+	Qualcomm Snapdragon XR2	Qualcomm Snapdragon XR2 Gen2
CPU			Octa-Core Kryo 585 (1 X 2.84 GHz, 3 X 2.42 GHz, 4 X 1.8 GHz)	Octa-Core Kryo 585 (1 X 2.84 GHz, 3 X 2.42 GHz, 4 X 1.8 GHz)	Octa-Core Kryo (1 X 3.19 GHz, 4 X 2.8 GHz, 3 X 2 GHz)
GPU			Adreno 650	Adreno 650	Adreno 740
Memory			12 GB LPDDR5	12 GB	8 GB

**Table 2:** Users’ list, with age, gender, and VR background experience.

User ID	Age	Gender	VR Exp (0–3)
1	29	Male	2
2	27	Male	3
3	26	Male	2
4	28	Male	2
5	35	Male	2
6	27	Female	0
7	29	Male	1
8	31	Male	0
9	27	Male	1
10	28	Female	1
11	30	Male	0
12	46	Male	0
13	25	Female	0
14	26	Female	1
15	33	Female	2
16	40	Female	2
17	30	Female	3
18	42	Male	2
19	50	Male	2
20	32	Female	0
21	29	Female	1
22	33	Male	0
23	46	Female	1
24	50	Female	0
25	37	Male	1
26	36	Female	1
27	40	Male	2
28	28	Female	1
29	36	Female	2
30	25	Female	0
31	26	Male	3
32	30	Male	3
33	31	Male	2
34	28	Female	0
35	40	Male	2
36	27	Female	1
37	30	Male	1
38	32	Male	0
39	29	Female	3
40	45	Female	2
41	47	Female	1
42	39	Female	2
43	33	Male	0
44	29	Male	1
45	34	Female	3
46	33	Female	1
47	39	Male	0
48	25	Male	2
49	32	Female	0
50	27	Female	3
51	35	Female	0
52	36	Male	1
53	37	Male	2
54	28	Male	0
55	29	Male	3
56	37	Female	1
57	33	Male	2
58	39	Male	1
59	28	Female	3
60	32	Male	0

**Table 3:** Mapping between the proposed tasks and concrete industrial workflows.

Task (s)	Industrial Workflow
Tasks 1–2 (near/far pick and place)	Retrieving components from a parts tray or from a distant shelf (assembly line, warehouse)
Tasks 3–4 (pick, rotate and insert)	Inserting a pin, tightening a screw, coupling a connector (mechanical assembly)
Task 5 (two-hand dynamics)	Handling large or articulated parts (e.g., placing a car door, assembling a tool)
Task 6 (button interaction)	Operating a control panel, entering numeric codes on an industrial keypad
Task 7 (teleporting)	Navigating large industrial sites (factory floor, power plant) for inspection or maintenance
Task 8 (pick and throw)	Sorting defective items into a reject bin, disposing of waste materials (quality control)
Task 9 (reading canvas at distance)	Reading instructions, safety warnings, or instrument displays from several meters away
Task 10 (interactive dynamics—catching falling spheres)	Intercepting falling components in high-speed assembly, picking moving items from a conveyor belt
Tasks 11–15 (qualitative)	Visual inspection of high-resolution models, auditory anomaly detection, ergonomic assessment, low-light operation

**Table 4:** Reference time for the first seven quantitative tasks.

Task	$t_{\mathrm{ref}}$ [s]
1	9.75
2	9.63
3	7.84
4	11.23
5	6.86
6	8.10
7	7.05

**Table 5:** Results of Mauchly’s test for the sphericity assumption and the corresponding corrections.

Task	*p*	GG-ε	Correction
1	0.071	0.883	None
2	<0.001	0.744	Greenhouse–Geisser
3	<0.001	0.635	Greenhouse–Geisser
4	0.020	0.863	Huynh–Feldt
5	0.030	0.859	Huynh–Feldt
6	<0.001	0.583	Greenhouse–Geisser
7	0.037	0.866	Huynh–Feldt
8	<0.001	0.824	Huynh–Feldt
9	<0.001	0.507	Greenhouse–Geisser
10	<0.001	0.796	Huynh–Feldt
11	<0.001	0.801	Huynh–Feldt
12	<0.001	0.773	Huynh–Feldt
13	<0.001	0.618	Greenhouse–Geisser
14	<0.001	0.770	Huynh–Feldt
15	0.006	0.845	Huynh–Feldt

**Table 6:** Results of RM-ANOVA: *p*-values and generalized $\eta^{2}$.

Task	*p*-Value	$\eta_{G}^{2}$
1	<0.001	0.296
2	<0.001	0.156
3	<0.001	0.223
4	<0.001	0.456
5	<0.001	0.238
6	<0.001	0.603
7	<0.001	0.253
8	<0.001	0.271
9	<0.001	0.131
10	<0.001	0.452
11	<0.001	0.149
12	<0.001	0.338
13	<0.001	0.406
14	<0.001	0.467
15	<0.001	0.129

**Table 7:** List of VR devices and their acronyms used throughout the tables.

Full Name	Acronym
HTC VIVE Pro 1	VP1
HTC VIVE Pro 2	VP2
VIVE XR Elite	XR-E
Meta Quest 3	MQ3
Meta Quest Pro	MQ-Pro

**Table 8:** Task 1—Near-field manipulation: pick and place. Tukey’s post hoc pairwise comparisons.

	MQ-Pro	MQ3	XR-E	VP2
VP1	0.206	<0.001	<0.001	0.15
VP2	0.970	0.075	<0.001	-
XR-E	<0.001	0.144	-	
MQ3	0.055	-		

**Table 9:** Task 2—Far-field manipulation: pick and place. Tukey’s post hoc pairwise comparisons.

	MQ-Pro	MQ3	XR-E	VP2
VP1	0.001	0.443	<0.001	0.531
VP2	<0.001	0.010	<0.001	-
XR-E	0.003	<0.001	-	
MQ3	0.073	-		

**Table 10:** Task 3—Near-field manipulation: pick, rotate and insert. Tukey’s post hoc pairwise comparisons.

	MQ-Pro	MQ3	XR-E	VP2
VP1	0.454	0.029	0.009	<0.001
VP2	<0.001	<0.001	<0.001	-
XR-E	0.058	0.677	-	
MQ3	0.236	-		

**Table 11:** Task 4—Far-field manipulation: pick, rotate and insert. Tukey’s post hoc pairwise comparisons.

	MQ-Pro	MQ3	XR-E	VP2
VP1	0.339	0.880	<0.001	<0.001
VP2	<0.001	<0.001	<0.001	-
XR-E	<0.001	0.013	-	
MQ3	0.160	-		

**Table 12:** Task 5—Two-hand dynamics. Tukey’s post hoc pairwise comparisons.

	MQ-Pro	MQ3	XR-E	VP2
VP1	0.213	<0.001	<0.001	0.175
VP2	0.974	<0.001	<0.001	-
XR-E	<0.001	0.874	-	
MQ3	<0.001	-		

**Table 13:** Task 6—Button interaction. Tukey’s post hoc pairwise comparisons.

	MQ-Pro	MQ3	XR-E	VP2
VP1	<0.001	<0.001	<0.001	0.298
VP2	<0.001	<0.001	<0.001	-
XR-E	<0.001	<0.001	-	
MQ3	0.003	-		

**Table 14:** Task 7—Teleporting. Tukey’s post hoc pairwise comparisons.

	MQ-Pro	MQ3	XR-E	VP2
VP1	0.672	<0.001	0.120	<0.001
VP2	<0.001	0.142	<0.001	-
XR-E	0.006	<0.001	-	
MQ3	0.024	-		

**Table 15:** Task 8—Dynamics: pick and throw. Tukey’s post hoc pairwise comparisons.

	MQ-Pro	MQ3	XR-E	VP2
VP1	<0.001	<0.001	<0.001	<0.001
VP2	0.004	0.002	0.928	-
XR-E	0.021	0.009	-	
MQ3	0.998	-		

**Table 16:** Task 9—Reading canvas at different distances. Tukey’s post hoc pairwise comparisons.

	MQ-Pro	MQ3	XR-E	VP2
VP1	0.012	0.054	0.416	0.180
VP2	0.358	0.236	<0.001	-
XR-E	<0.001	<0.001	-	
MQ3	0.980	-		

**Table 17:** Task 10—Interactive dynamics (catching falling spheres). Tukey’s post hoc pairwise comparisons.

	MQ-Pro	MQ3	XR-E	VP2
VP1	0.021	0.092	<0.001	0.232
VP2	<0.001	<0.001	<0.001	-
XR-E	<0.001	<0.001	-	
MQ3	0.994	-		

**Table 18:** Task 11—Hi-res model inspection. Tukey’s post hoc pairwise comparisons.

	MQ-Pro	MQ3	XR-E	VP2
VP1	0.004	0.100	0.010	0.024
VP2	<0.001	<0.001	<0.001	-
XR-E	1.000	0.745	-	
MQ3	0.254	-		

**Table 19:** Task 12—Sound listening. Tukey’s post hoc pairwise comparisons.

	MQ-Pro	MQ3	XR-E	VP2
VP1	<0.001	<0.001	<0.001	0.143
VP2	<0.001	<0.001	<0.001	-
XR-E	0.343	0.986	-	
MQ3	0.736	-		

**Table 20:** Task 13—Mid-exposure tolerability Tukey’s post hoc pairwise comparisons.

	MQ-Pro	MQ3	XR-E	VP2
VP1	0.542	1.000	<0.001	0.101
VP2	0.623	0.145	<0.001	-
XR-E	<0.001	<0.001	-	
MQ3	0.512	-		

**Table 21:** Task 14—Ergonomics and comfort in wearing. Tukey’s post hoc pairwise comparisons.

	MQ-Pro	MQ3	XR-E	VP2
VP1	0.770	<0.001	<0.001	<0.001
VP2	0.036	0.821	<0.001	-
XR-E	<0.001	<0.001	-	
MQ3	<0.001	-		

**Table 22:** Task 15—Low-light environment sensibility. Tukey’s post hoc pairwise comparisons.

	MQ-Pro	MQ3	XR-E	VP2
VP1	0.172	0.001	<0.001	1.000
VP2	0.006	<0.001	<0.001	-
XR-E	0.070	0.308	-	
MQ3	0.762	-		

## Data Availability

Disaggregated data available upon request.
